# A multicenter, matched case–control analysis comparing burden of illness among patients with tuberous sclerosis complex related epilepsy, generalized idiopathic epilepsy, and focal epilepsy in Germany

**DOI:** 10.1186/s42466-024-00323-6

**Published:** 2024-05-30

**Authors:** Lisa Lappe, Christoph Hertzberg, Susanne Knake, Markus Knuf, Felix von Podewils, Laurent M. Willems, Stjepana Kovac, Johann Philipp Zöllner, Matthias Sauter, Gerhard Kurlemann, Thomas Mayer, Astrid Bertsche, Klaus Marquard, Sascha Meyer, Hannah Schäfer, Charlotte Thiels, Bianca Zukunft, Susanne Schubert-Bast, Jens-Peter Reese, Felix Rosenow, Adam Strzelczyk

**Affiliations:** 1 Goethe-University Frankfurt, Epilepsy Center Frankfurt Rhine-Main, Department of Neurology, University Hospital Frankfurt, Theodor-Stern-Kai 7, 60596 Frankfurt am Main, Germany; 2 Center for Personalized Translational Epilepsy Research (CePTER), Goethe-University Frankfurt, Frankfurt am Main, Germany; 3 Department of Neuropediatrics, Vivantes Klinikum Neukölln, Berlin, Germany; 4 Epilepsy Center Hessen and Department of Neurology, Philipps-University Marburg, Marburg, Germany; 5 Department of Pediatrics, Klinikum Worms, Worms, Germany; 6 Department of Pediatrics, University Medicine Mainz, Mainz, Germany; 7 Department of Neurology, Epilepsy Center, University Medicine Greifswald, Greifswald, Germany; 8 Department of Neurology with Institute of Translational Neurology, University Münster, Münster, Germany; 9 Klinikum Kempten, Klinikverbund Allgäu, Kempten/Allgäu, Germany; 10 St. Bonifatius Hospital, Lingen, Germany; 11 Epilepsy Center Kleinwachau, Radeberg, Germany; 12 Department of Neuropediatrics, University Hospital for Children and Adolescents, Greifswald, Germany; 13 Department of Pediatric Neurology, Psychosomatics and Pain Management, Klinikum Stuttgart, Stuttgart, Germany; 14 Department of General Pediatrics and Neonatology, Franz-Lust Klinik für Kinder und Jugendliche, Karlsruhe, Germany; 15 Department of Nephrology, Klinikum rechts der Isar, Technische Universität München, Munich, Germany; 16 Division of Nephrology, Medizinische Klinik und Poliklinik IV, Klinikum der LMU München - Innenstadt, Munich, Germany; 17 Department of Neuropediatrics and Pediatric Epileptology, University Hospital of Ruhr University Bochum, Bochum, Germany; 18 Department of Nephrology and Internal Intensive Care, Charité - University Medicine Berlin, Berlin, Germany; 19 Goethe-University Frankfurt, Department of Neuropediatrics, University Hospital Frankfurt, Frankfurt am Main, Germany; 20 Institute of Clinical Epidemiology and Biometry, University of Würzburg, Würzburg, Germany

**Keywords:** Seizure, Anti-seizure medication, Anticonvulsants, Everolimus, Costs, Direct costs, Indirects costs, Quality of life, Depression, Stigma

## Abstract

**Background:**

Depending on the underlying etiology and epilepsy type, the burden of disease for patients with seizures can vary significantly. This analysis aimed to compare direct and indirect costs and quality of life (QoL) among adults with tuberous sclerosis complex (TSC) related with epilepsy, idiopathic generalized epilepsy (IGE), and focal epilepsy (FE) in Germany.

**Methods:**

Questionnaire responses from 92 patients with TSC and epilepsy were matched by age and gender, with responses from 92 patients with IGE and 92 patients with FE collected in independent studies. Comparisons were made across the main QoL components, direct costs (patient visits, medication usage, medical equipment, diagnostic procedures, ancillary treatments, and transport costs), indirect costs (employment, reduced working hours, missed days), and care level costs.

**Results:**

Across all three cohorts, mean total direct costs (TSC: €7602 [median €2620]; IGE: €1919 [median €446], *P* < 0.001; FE: €2598 [median €892], *P* < 0.001) and mean total indirect costs due to lost productivity over 3 months (TSC: €7185 [median €11,925]; IGE: €3599 [median €0], *P* < 0.001; FE: €5082 [median €2981], *P* = 0.03) were highest among patients with TSC. The proportion of patients with TSC who were unemployed (60%) was significantly larger than the proportions of patients with IGE (23%, *P* < 0.001) or FE (34%, *P* = *P* < 0.001) who were unemployed. Index scores for the EuroQuol Scale with 5 dimensions and 3 levels were significantly lower for patients with TSC (time-trade-off [TTO]: 0.705, visual analog scale [VAS]: 0.577) than for patients with IGE (TTO: 0.897, VAS: 0.813; *P* < 0.001) or FE (TTO: 0.879, VAS: 0.769; *P* < 0.001). Revised Epilepsy Stigma Scale scores were also significantly higher for patients with TSC (3.97) than for patients with IGE (1.48, *P* < 0.001) or FE (2.45, *P* < 0.001). Overall Quality of Life in Epilepsy Inventory-31 items scores was significantly lower among patients with TSC (57.7) and FE (57.6) than among patients with IGE (66.6, *P* = 0.004 in both comparisons). Significant differences between patients with TSC and IGE were also determined for Neurological Disorder Depression Inventory for Epilepsy (TSC: 13.1; IGE: 11.2, *P* = 0.009) and Liverpool Adverse Events Profile scores (TSC: 42.7; IGE: 37.5, *P* = 0.017) with higher score and worse results for TSC patients in both questionnaires.

**Conclusions:**

This study is the first to compare patients with TSC, IGE, and FE in Germany and underlines the excessive QoL burden and both direct and indirect cost burdens experienced by patients with TSC.

**Supplementary Information:**

The online version contains supplementary material available at 10.1186/s42466-024-00323-6.

## Key points

• This is the first analysis to compare QoL and direct/indirect cost burden among patients with TSC, IGE, and FE in Germany.

• Generic QoL for patients with TSC was significantly lower than for patients with IGE and FE.

• Care grade and disability cards were more frequently obtained by patients with TSC than by patients in the other cohorts.

• Drug treatment costs were the highest direct cost component for patients with TSC.

• More patients with TSC than with IGE or FE were unemployed, and the mean indirect productivity costs were highest for the TSC cohort.

**Supplementary Information:**

The online version contains supplementary material available at 10.1186/s42466-024-00323-6.

## Background

Tuberous sclerosis complex (TSC) is a rare genetic disorder with a widely variable and multiorgan expression pattern and an estimated incidence of 1:6760 to 1:13,520 live births in Germany [1]; however, the true incidence may be higher, as substantial phenotypic variability may result in undetected cases [2]. Loss-of-function mutations in one of two tumor suppressor genes, *TSC1* and *TSC2*, lead to the overactivation of the mechanistic target of rapamycin (mTOR) pathway, dysregulation of cell proliferation, and the formation of benign tumors in multiple organ systems, including the brain [3, 4]. Numerous individuals with TSC suffer from structural epilepsy caused by the formation of cortical tubers or other cortical malformations. Early onset epilepsy often initially presents as focal seizures, accompanied by infantile spasms. However, patients with TSC can present with almost all seizure types, including tonic, atonic, and tonic-clonic seizures [5–8].

Studies evaluating the direct and indirect economic burden borne by patients with epilepsy have reported different costs associated with resource consumption for different seizure types or epilepsy syndromes [9]. Past studies reported higher resource consumption among patients with focal epilepsy (FE), whereas individuals with idiopathic generalized epilepsy (IGE), which has a better prognosis than FE, reported lower costs [10–12]. Multiple studies have evaluated the direct and indirect economic and quality of life (QoL) burdens experienced by patients with TSC [13–17]. However, the economic and QoL impacts experienced in Germany by patients with TSC related epilepsy have not previously been compared with patients experiencing other epilepsy syndromes, such as IGE and FE.

This study was conducted to compare the direct and indirect costs and QoL impacts among patients with TSC and epilepsy, IGE and FE. Patients with TSC related epilepsy were age- and gender-matched with patients with IGE and FE in Germany, using datasets derived from similar questionnaires based studies featuring common components and data collection instruments.

## Methods

### Disease cohort datasets and matching

Studies used for this analysis have explored the cost of illness and QoL among patients with TSC [16, 17] and epilepsy syndromes [10, 11] in Germany using similarly structured retrospective questionnaires to evaluate direct and indirect costs, QoL, and psychometric impacts.

The German multicenter cohort TSC study was completed in 2019, enrolling 192 adults with TSC treated by 14 centers throughout Germany and through the German TSC patient advocacy group (Tuberöse Sklerose Deutschland e.V., Wiesbaden, Germany). The TSC study included patients with different organ manifestations, such as epilepsy, structural brain defects, and heart and circulatory system disorders [16, 17]. The present analysis included only those patients with an identified epilepsy independent of additional organ manifestations.

The Epi2020 study was completed in 2020, enrolling 486 adult patients with epilepsy treated by four different epilepsy centers: Frankfurt am Main, Greifswald, Marburg, and Münster. The study included patients irrespective of seizure severity, duration of illness, or epilepsy syndrome [10, 11, 18–22]. In the present analysis, only epilepsy patients diagnosed with either IGE or FE were included.

Each patient with TSC related epilepsy was matched with one patient with IGE and one patient with FE based on age and sex, with a tolerated range of ± 5 years. Each cohort (TSC, IGE, and FE) generated for the comparative analysis included 92 patients.

Both studies received ethics approval and were registered at the German Clinical Trials Register (TSC study: DRKS00016045; Epi2020 study: DRKS00022024). All patients provided informed consent, meaning that individuals under legal guardianship due to intellectual disability were not included. The classification of seizure types and epilepsy syndromes was based on definitions proposed by the International League Against Epilepsy (ILAE) [23, 24]. The diagnostic criteria for TSC were based on the latest recommendations established by the 2012 International TSC Consensus Conference [25]. The Strengthening the Reporting of Observational Studies in Epidemiology (STROBE) guidelines were followed [26].

### Cost calculations

Cost analysis was conducted to calculate and compare both direct and indirect costs experienced by patients with TSC who suffer from epilepsy with those experienced by patients with IGE or FE. A bottom-up approach was applied to evaluate the overall economic burden associated with each disease from a societal perspective of the statutory health insurer “Gesetzliche Krankenversicherung” (GKV).

Direct costs included healthcare resource use, such as inpatient and outpatient hospital visits, rehabilitation, drug treatment (anti-seizure medication [ASMs], other prescribed drugs, and mTOR inhibitors), healthcare professional visits, ancillary treatments, diagnostic costs, emergency transportation, and medical aids. Costs for inpatient and outpatient visits, health care professional visits, ancillary therapies, and diagnostic tests were calculated and standardized as described by Bock et al. [27] using physician fee scales (Einheitlicher Bewertungsmaßstab) [28], whereas drug treatment costs were computed based on the Drug Prescription Report 2020 [29]. The quantification of ASM drug load was presented through both the mean and median values of the number of ASMs and mean and median Defined Daily Dose (DDD). The DDD serves as a proxy for the assumed average maintenance dose per day [30].

Additionally, care grade costs associated with care levels 1–5 were compared. To ensure comparability, the reported costs for patients with TSC in 2019 were adjusted for inflation to reflect the expected costs in 2020 using the consumer price index for Germany in 2020.

Indirect (productivity) costs were calculated for individuals who reported an inability to work, unemployment, working part-time work or reduced hours, days off, early retirement, homemaker, and receiving a pension for disability workshops. Indirect costs were computed using the human capital approach for patients of working age (< 67 years) [10] using a mean annual gross wage of €47,700 (€3975 per month; €131 per calendar day) in 2020.

### Questionnaires and instruments

Data for this comparative analysis were retrieved from retrospective questions spanning the previous 3 months. Questionnaires included six instruments: EuroQol-5 dimensions-3 levels inventory (EQ-5D-3L), including the EuroQol self-rated Visual Analog Scale (EQ-VAS); the Quality of Life in Epilepsy Inventory-31 items (QOLIE-31); the Neurological Disorder Depression Inventory for Epilepsy (NDDI-E); the Liverpool Adverse Events Profile (LAEP); the revised Epilepsy Stigma Scale (rESS); and the Seizure Worry Scale.

The EQ-5D-3L measures generic QoL, including mobility, self-care, usual activities, pain/discomfort, and anxiety/depression, using a 3-level Likert scale (1 indicates no problems, 2 indicates mild problems, 3 indicates massive problems). The responses are transformed into continuous summary indexes, according to the German value set, such that each combination is weighted with prespecified values using both the time-trade-off (TTO) and VAS methods. A summary index score of 1 indicates perfect health, 0 indicates health status equivalent to death, and negative values indicate health status worse than death [31]. Raw, self-rated VAS scores were reported alongside the EQ-5D summary index score, ranging from 0 (worst health imaginable) to 100 (best health imaginable) [31].

The QOLIE-31 measures health-related QoL across the seven dimensions: overall QoL, seizure worry, emotional well-being, energy/fatigue, cognitive function, medication effects, and social function [32]. A separate VAS, analogous to the raw VAS for EQ-5D, is available for the QOLI-31. QOLIE-31 scores were calculated using the manual, ranging from 0 (worst disease-related health) to 100 (best disease-related health) [32].

The NDDI-E assesses depression among patients with epilepsy using six 4-level Likert items regarding the frequency of common depressive symptoms, such as feeling guilty, frustrated, or weary of life. An aggregated value ≥ 14 points (range 6–24 points) suggests depressive mood [33, 34].

Therapy-related adverse events were measured using the LAEP, which includes twenty 4-level Likert (1–4) items; a cutoff score of 35 points (range 19–76) indicates relevant therapy-related adverse events [35].

The rESS compromises three questions regarding epilepsy-related stigmatization [36] (‘I feel that some people are uncomfortable with me,’ ‘I feel some people treat me like an inferior person,’ and ‘I feel some people would prefer to avoid me’) using a 4-level-Likert scale (0–3), with a range from 0 to 9 points. A total score of 0 indicates no stigma, scores of 1–6 indicate mild or moderate stigma, and scores of 7–9 indicate high stigma [37]. The Seizure Worry Scale, a two-item instrument using a 4-level-Likert-scale (0–3) as well, with a range from 0 to 6 points, indicates patients’ worries associated with epileptic seizures [38].

### Statistical analysis

Statistical analyses were performed using IBM SPSS Statistics version 28.0.1.1 (SPSS Inc., Chicago, IL, U.S.A). For continuous variables, the means, medians, standard deviations (SDs), minima, maxima, and ranges were calculated for each cohort. Because most cost variables are highly skewed, a 95% confidence interval was calculated using the bootstrap method, according to the bias-corrected accelerated approach [39, 40]. Differences in continuous variables between three cohorts were tested for significance using a parametric analysis of variance for normally distributed data and a non-parametric Kruskal–Wallis test for data that were not normally distributed. The null hypothesis was that no significant differences existed between groups. For categorical variables, frequencies and percentages were calculated for each cohort. Differences in the distribution of categorical variables between cohorts were tested for significance using a two-tailed Pearson’s Chi-square test, followed by hypothesis-based, pairwise, Pearson’s Chi-square tests separately comparing the TSC cohort with both the IGE and FE cohorts. The null hypothesis was that no significant differences existed between cohorts. Differences between the TSC, IGE, and FE cohorts were considered significant at *P* < 0.05. A Bonferroni correction for multiple tests was applied.

## Results

### Demographic factors and clinical characteristics

The cohorts were well-matched for age and sex, with no statistical differences across cohorts (Additional File 1). The mean ages for the TSC, IGE, and FE cohorts were 32.0 years, 31.8 years, and 32.0 years, respectively. The sex distribution was consistent across all cohorts, with 60 female patients and 32 male patients in each group.

Among patients with TSC, the first seizure was reported at a mean age of 1.9 years (median: 0 years, SD: 4.6 years), which was significantly earlier than the age of first seizure reported for patients with IGE (mean: 16.9 years, median: 16.0 years, SD: 8.9 years; *P* < 0.001) and FE (mean: 15.9 years, median: 14.0 years, SD: 10.9 years, *P* < 0.001). Among those with FE, 46 had temporal lobe epilepsy, 10 had frontal lobe epilepsy, and 36 epilepsy from other or multiple lobes. Among those with IGE, 35 had Juvenile Myoclonic Epilepsy, 8 Childhood or Juvenile Absence Epilepsy and 49 Generalized Tonic–Clonic Seizures Alone or undetermined IGE syndrome. TSC was diagnosed at a mean age of 8.0 years (median: 1.0 years, SD: 13.2 years). In addition to epilepsy, the majority of patients with TSC in our cohort suffered from multiple organ manifestations, including 87 (95%) reporting skin manifestations, 76 (83%) reporting neurological disorders other than epilepsy, 68 (74%) reporting kidney and urinary tract manifestations, and 45 (49%) reporting heart and circulatory system disorders.

**Fig. 1:**
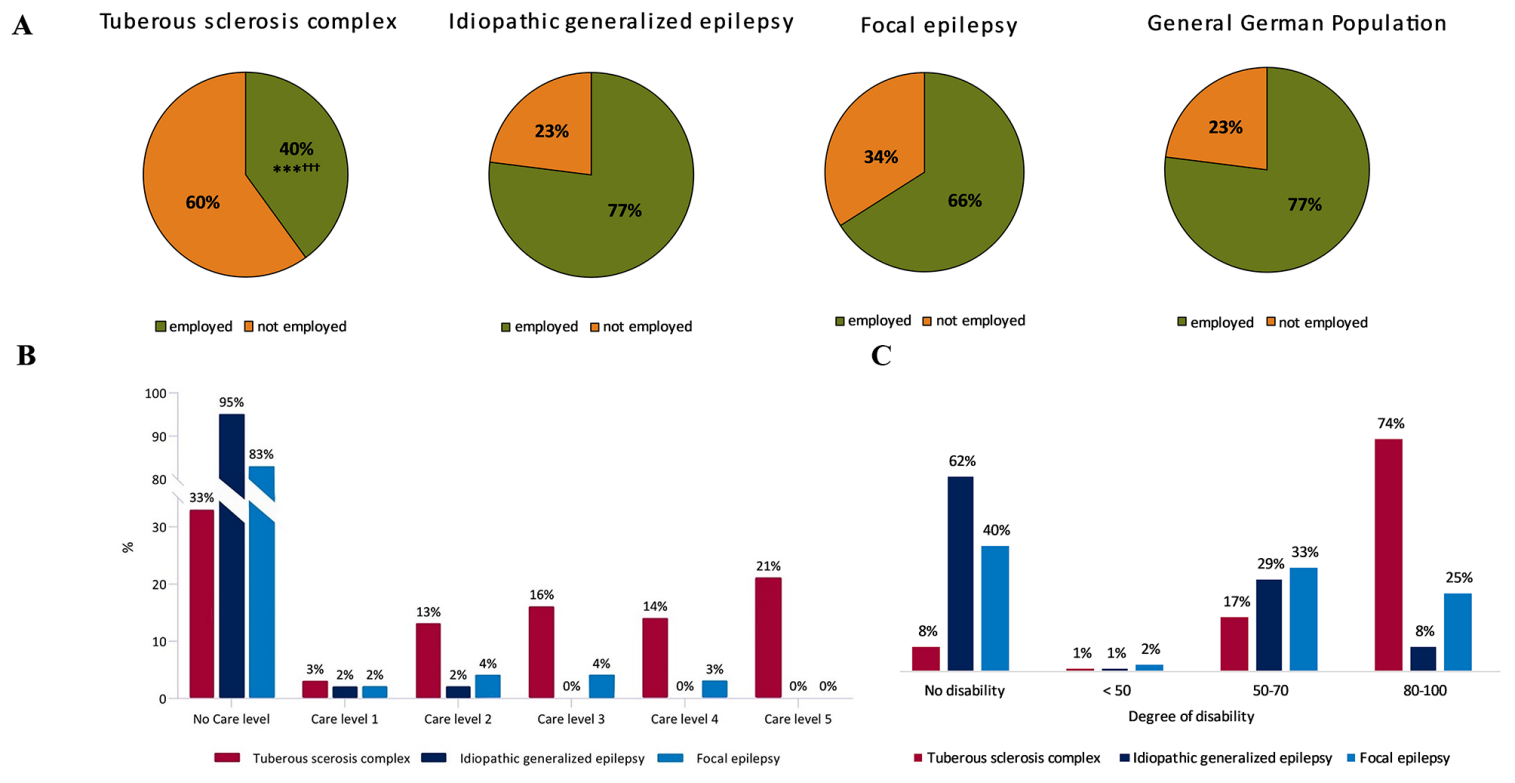
**A**. The proportions of patients reporting employment (full-time, part-time/hour reduction, vocational training) in the tuberous sclerosis complex (TSC; missing *n* = 7), idiopathic generalized epilepsy (IGE), and focal epilepsy (FE, missing *n* = 2) cohorts and general German population *P < 0.05, **P < 0.01, and ***P < 0.001 between the TSC and IGE cohorts; †*P* < 0.05, ††*P* < 0.01, and †††*P* < 0.001 between the TSC and FE cohorts **B**. Percentage of patients in each cohort (*n* = 92) receiving the indicated care level. Using the Chi-square test, significant differences were identified between the proportion of patients with TSC with a care grade and the proportions of patients with IGE (missing *n* = 1; *P* < 0.001) and FE (missing *n* = 3; *P* < 0.001) with a care grade **C**. Percentage of patients in each cohort (*n* = 92) with some degree of disability. Using the Chi-square test, significant differences were identified between the proportion of patients with TSC with a disability card (degree of disability ≥ 50) and the proportions of patients with IGE (*P* < 0.001) and FE (*P* < 0.001) with a disability card

Patients with TSC (51/85, 60%) more frequently reported unemployment (with employment defined as working full-time, part-time, or reduced hours or participating in vocational training) than patients with IGE (21/92, 23%) or FE (31/90, 34%; *P* < 0.001 in both comparisons; Fig. 1A). A higher proportion of patients with IGE (50/92, 54%) reported full-time employment than patients with TSC (21/85, 25%; *P* < 0.001).

The proportion of patients with TSC with a disability card (degree of disability ≥ 50) was significantly higher (*n* = 84, 91%) than the proportions of patients with IGE (*n* = 34, 37.0%; *P* < 0.001) or FE (*n* = 53, 58%; *P* < 0.001; Fig. 1C).

In addition, patients with TSC were significantly more likely to have a care grade than patients with IGE or FE (*P* < 0.001 for both comparisons), and the TSC cohort had higher care grades overall, especially care levels 3–5 (Fig. 1B).

### Seizure frequency

In the TSC cohort, 35 patients (38%) had experienced no seizures in the past year compared with 42 patients (46%) in the IGE cohort and 29 patients (32%) in the FE cohort. Patients in all three cohorts reported experiencing seizures on a yearly, half-yearly, monthly, weekly, or daily basis (Additional File 2). More patients in the TSC cohort reported daily, weekly or monthly seizures (46/92) than patients in the IGE cohort (21/92, *P* > 0.001), whereas more patients in the IGE cohort (63/92) reported seizures every 6 months or less frequently. No significant differences in seizure frequency were identified between the TSC and FE (39/92) cohorts (Additional File 2).

### Direct healthcare resource costs and care grade costs

Comparing direct costs across cohorts revealed total mean direct costs of €7602 (median: €2620, SD: €10,090) for the TSC cohort, €1919 (median: €446, SD: €3564) for the IGE cohort, and €2598 (median: €892, SD: €5229) per three months for the FE cohort. Patients with TSC had significantly higher total direct costs than patients with IGE or FE (*P* < 0.001 for both comparisons; Table 1).

**Table 1:** Total mean and median direct costs per disease cohort over three months measured in Euros (€)

		TSC (*N* = 92)		IGE (*N* = 92)		FE (*N* = 92)		*P*-value	*P*-value	*P*-value	95% CI^(a)^		
		Mean	Median	Mean	Median	Mean	Median	TSC/IGE	TSC/FE	FE/IGE	TSC	IGE	FE
Total cost													
Medical aids**††		63.4	0	0.87	0	5.1	0	0.009	0.009	1.00	22.7–114.2	0–2.1	0–14.6
Hospital and rehabilitation costs		1443.5	0	1125.6	0	1478.1	0	1.00	1.00	1.00	647.5–2546.0	618.4–1678.0	790.2–2329.6
	Rehabilitation	84.8	0	0	0	93.5	0	0.65	1.00	0.65	0–212.6	N/A	0–217.3
	Inpatient	1358.6	0	1125.6	0	1384.7	0	1.00	1.00	1.00	567.2–2564.6	587.5–1758.4	703.4–2315.3
Ancillary treatments ***†††		165.3	0	12.5	0	37.4	0	< 0.001	< 0.001	0.97	104.2–237.1	1.0–27.9	13.3–66.0
Diagnostic tests***†		169.3	61.6	66.9	22.8	79.1	31.5	< 0.001	0.01	1.00	118.6–224.0	43.6–94.7	57.3–102.0
Outpatient costs ***†††		494.0	308.7	193.7	48.6	243.8	70.3	< 0.001	< 0.001	0.73	386.1–617.6	135.0–261.2	172.6–327.9
	Doctor outpatient costs***†††	273.1	179.3	92.1	48.6	120.1	48.6	< 0.001	< 0.001	1.00	222.1–334.0	68.9–119.7	84.3–165.4
	Outpatient hospital costs	220.9	0	101.6	0	123.7	0	0.08	0.28	1.00	145.9–306.9	56.0–153.9	73.5–181.2
Total drug treatment costs***		5187.9	584.0	447.8	191.6	695.9	439.9	< 0.001	0.34	< 0.001	3704.1–6769.4	296.9–639.1	550.1–851.8
	ASM costs ††	658.3	145.9	416.5	161.0	661.7	384.5	1.00	< 0.001	< 0.001	333.3–1177.8	260.7–615.0	520.7–831.9
	Costs for other prescribed drugs***†††	143.6	33.7	31.3	0	34.2	0	< 0.001	< 0.001	0.35	81.1–228.0	13.8–52.4	19.2–50.9
	mTOR inhibitor costs	4386.0	0	N/A	N/A	N/A	N/A	N/A	N/A	N/A	3018.9–5829.1	N/A	N/A
Transport costs		78.3	0	71.7	0	58.7	0	1.00	1.00	1.00	29.6–140.2	28.6–125.0	13.5–115.0
Total direct costs ***†††		7601.7	2619.8	1919.1	446.1	2598.1	891.7	< 0.001	< 0.001	0.07	5712.5–9797.0	1294.4–2621.3	1773.7–3627.5
Care grade cost***†††		1257.1	1635.0	20.6	0	183.5	0	< 0.001	< 0.001	0.47	1045.5–1479.0	0.00–49.5	94.4–291.1

*P < 0.05, **P < 0.01, and ***P < 0.001 between patients with TSC and IGE; †*P* < 0.05,††*P* < 0.01, and †††*P* < 0.001 between patients with TSC and FE

^a)^ 95% confidence intervals were calculated for costs using the bias-corrected accelerated (BCa) bootstrap approach

TSC: tuberous sclerosis complex; IGE: idiopathic generalized epilepsy; FE: focal epilepsy; ASM: anti-seizure medications; mTOR: mechanistic target of rapamycin; CI: confidence interval; N/A: not applicable

The highest direct costs for patients with TSC resulted from drug treatments. Total drug treatment costs, including ASMs, other prescribed drugs, and mTOR inhibitors, were significantly higher for the TSC cohort (mean: €5188, median: €584, SD: €7815) than for the IGE cohort (mean: €448, median: €192, SD: €966; *P* < 0.001). Total drug treatment costs for the TSC cohort were also higher than for the FE cohort (mean: €696, median: €440, SD: €812), but this difference was not significant (*P* = 0.34).

ASM treatment costs were nearly equal between patients with TSC (mean: €658.3, median: €145.9) and FE (mean: €661.7, median: €384.5), whereas patients with IGE had significantly lower ASM treatment costs (mean: €416, median: €161) than patients with FE (*P* = 0.001).

No significant differences in the mean numbers of ASMs used were observed between the three cohorts (TSC: 1.73; IGE 1.54; FE: 1.86). We observed significant differences in the ASM drug load measured by DDD with the highest mean DDD of 3.09 (median: 3.0, SD: 1.82) in the FE cohort as compared to TSC 2.37 (median: 1.73, SD: 1.62; *P* = 0.034) and IGE 1.92 (median: 1.75, SD: 1.3; *P* < 0.001), with a no difference between TSC and IGE.

**Fig. 2:**
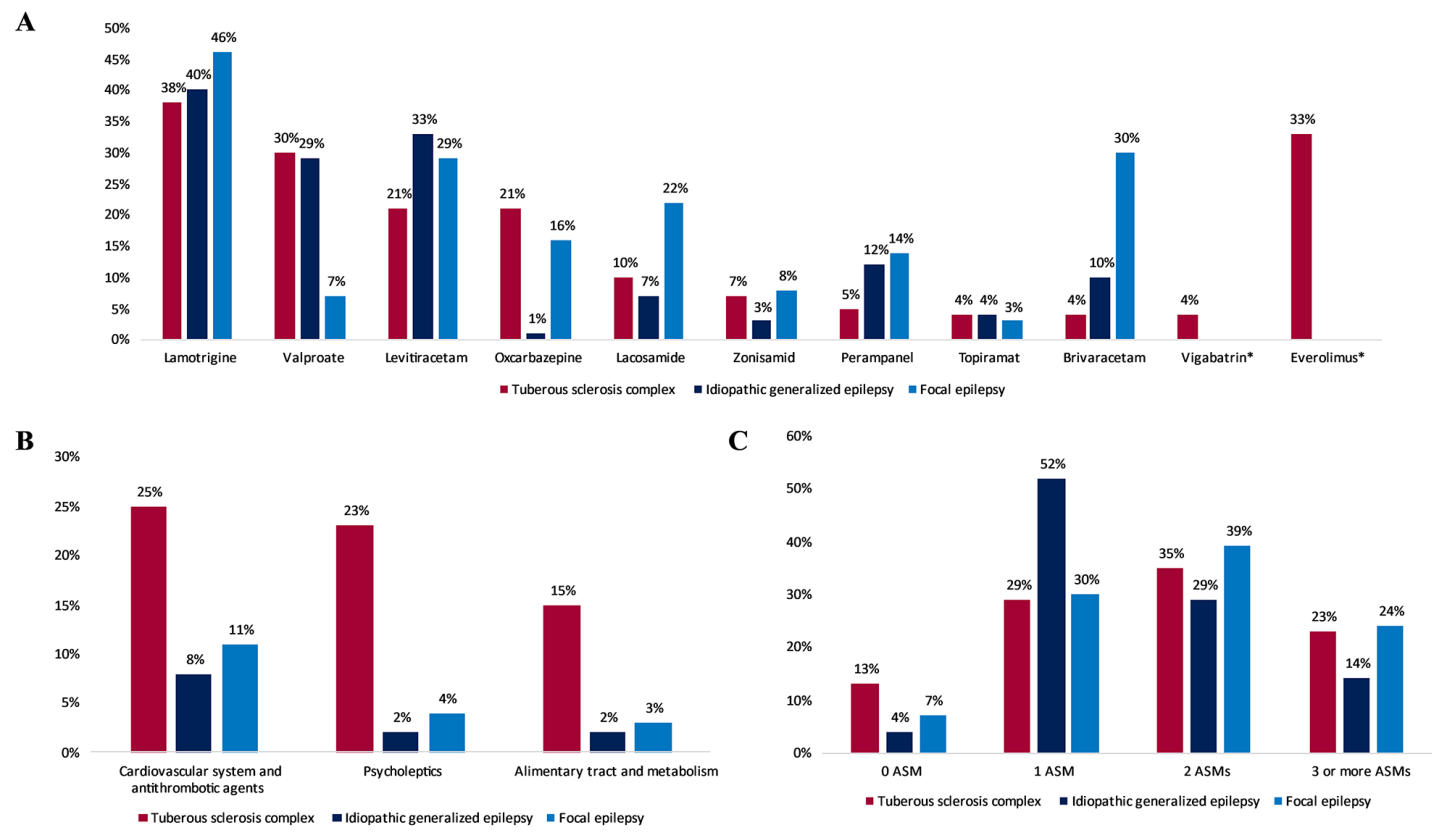
(**A**) Proportion of patients taking the indicated anti-seizure medications (ASM) by disease cohort (*n* = 92 per cohort). * drugs intended only for patients with TSC (**B**) Proportion of patients from each cohort (*n* = 92) taking at least one medication belonging to the indicated groups of prescribed drugs. Using the Chi-square test, significant differences were identified between the proportions of patients with tuberous sclerosis complex (TSC) and the proportions of patients with idiopathic generalized epilepsy (IGE; *P* = 0.006) taking cardiovascular and antithrombotic drugs, between the proportions of patients with TSC and the proportions of patients with IGE (*P* < 0.001) and focal epilepsy (FE; *P* < 0.001) taking psycholeptics, and between the proportions of patients with TSC and the proportions of patients with IGE (*P* = 0.01) and FE (*P* = 0.03) taking drugs for the alimentary tract and metabolism (**C**) The proportions of patients from each cohort (*n* = 92) taking the indicated number of daily anti-seizure medications (ASM)

The most frequently prescribed ASM for all three cohorts was lamotrigine (TSC: *n* = 35, 38%; IGE: *n* = 37, 40%; FE: *n* = 42, 46%). The next most frequently prescribed ASMs in the TSC group were valproate (*n* = 28, 30%), levetiracetam and oxcarbazepine (both *n* = 19, 21%), and lacosamide (*n* = 9, 10%; Fig. 2A). At least 2 ASMs were reported by 58% of patients with TSC, 43% of patients with IGE, and 63% of patients with FE, with no significant difference between cohorts (Fig. 2C).

Patients with TSC were associated with the highest costs for other prescribed drugs (mean: €144, median: €34, SD: €388) over the 3-month study period compared with patients with IGE (mean: €31, median: €0, SD: €105; *P* < 0.001) and FE (mean: €34, median: €0 SD: €77; *P* < 0.001), particularly drugs for the cardiovascular system and antithrombosis (e.g., antihypertensive drugs, lipid-modifying agents, platelet aggregation inhibitors), psycholeptics, and drugs for the alimentary tract and metabolism (e.g., diabetes, enzyme substitution). Details on the use of other prescribed drugs according to the Anatomical Therapeutic Chemical (ATC) classification system which indicate the organ or system on which they act and their therapeutic, pharmacological and chemical properties is presented in Additional File 3. A significantly higher proportion of patients with TSC reported at least one prescribed cardiovascular or antithrombotic drug (23/92, 25%) than patients with IGE (7/92, 8%; *P* = < 0.001). A significantly higher proportion of patients with TSC reported psycholeptic use (21/92, 23%) than both other cohorts (IGE: 2/92, 2%; FE: 4/92, 4%; *P* < 0.001 for both comparisons). A significantly higher proportion of patients with TSC reported alimentary tract and metabolism drugs than the other cohorts (TSC: 14/92, 15%; IGE: 2/92, 2%; FE 3/92, 3%; TSC vs. IGE: *P* = 0.01; TSC vs. FE: *P* = 0.03; Fig. 2B).

Because TSC results in mTOR pathway overactivation, patients with TSC experienced additional drug treatment costs due to mTOR inhibitor use (mean: €4386, median: €0, SD: €7452) during the 3-month study period, whereas patients with IGE and FE did not use mTOR inhibitors. mTOR inhibitors were reported by 31 patients with TSC (everolimus, *n* = 30; sirolimus *n* = 1), primarily to treat multiple organ manifestations, including 21 patients using mTOR inhibitors for angiomyolipoma, 17 patients for epilepsy, 15 patients for subependymal giant cell astrocytoma, and 6 patients for skin manifestation (multiple treatment targets possible).

No significant differences in mean overall hospitalization costs or costs due to epilepsy-related hospital stays were observed between cohorts. Eighteen patients with TSC reported a total of 23 inpatient hospital admissions with a mean duration of 2.2 days during the 3-month period, 21 of which were TSC-related and 8 of which were epilepsy-related. No significant differences in the mean duration of overall inpatient hospital admissions (TSC, 2.2 days; IGE, 1.8 days; FE, 2.6 days) or the mean duration of epilepsy-related hospital admissions (TSC, 0.4 days; IGE, 1.7 days; FE, 1.6 days) were observed among cohorts. Of the 20 hospital stays in the IGE cohort, 18 were epilepsy-related, and of the 28 hospital stays in the FE cohort, 20 were epilepsy-related. The most common reasons for epilepsy-related hospitalization were diagnostic procedures (TSC, *n* = 3; IGE, *n* = 4; FE, *n* = 8), seizures (TSC, *n* = 2; IGE, *n* = 3; FE, *n* = 5), and ASM changes (TSC, *n* = 0; IGE, *n* = 8; FE, *n* = 1). In addition, two patients with TSC and two patients with FE reported visiting an inpatient rehabilitation center.

Patients with TSC generated significantly higher outpatient costs (mean: €494, median: €309, SD: €570) than patients with IGE (mean: €194, median: €49, SD: €315; *P* < 0.001) or FE (mean: €244, median: €70, SD: €383; *P* < 0.001). Outpatient hospital stays were the highest outpatient costs, followed by consultations with neurologists. At least one outpatient consultation was reported by 85 patients with TSC (92%), 61 patients with IGE (66%), and 72 patients with FE (78%) in the past 3 months.

Costs related to ancillary treatments, such as physiotherapy, occupational therapy, and speech therapy, were significantly higher in the TSC cohort (mean: €165, median: €0, SD: €326) than in the IGE (mean: €13, median: €0, SD: €67; *P* < 0.001) or FE cohorts (mean: €37, median: €0, SD: €140; *P* < 0.001). At least one ancillary treatment was reported by 34 patients with TSC (37%), 5 patients with IGE (5%), and 10 patients with FE (11%), with physiotherapy and occupational therapy being the most frequent (Table 2).

**Table 2:** Mean numbers of specialist visits among the disease cohorts

	TSC			IGE			FE			*P*-value	*P*-value	*P*-value
	Mean	Median	SD	Mean	Median	SD	Mean	Median	SD	TSC vs. IGE	TSC vs. FE	FE vs. IGE
GP visits ***^†††^	1.90	1	2.61	0.53	0	1.22	0.74	0	1.37	< 0.001	< 0.001	0.72
Neurologist visits	1.25	1	1.68	1.01	1	1.02	1.33	1	2.26	1.00	1.00	1.00
Psychiatrist visits**^††^	0.57	0	1.48	0.43	0	1.92	0.36	0	1.46	0.005	0.01	1.00
Physiotherapy visits ***^†††^	3.23	0	6.30	0.17	0	1.05	0.46	0	2.77	< 0.001	< 0.001	1.00
Speech therapy visits**^†^	0.93	0	3.40	0	0	0	0.11	0	1.04	0.006	0.025	1.00
Occupational therapy visits***^†^	1.68	0	4.74	0.09	0	0.83	0.39	0	1.88	0.001	0.019	1.00

*P < 0.05, **P < 0.01, and ***P < 0.001 between patients with TSC and IGE; †*P* < 0.05,††*P* < 0.01, and †††*P* < 0.001 between patients with TSC and FE. Abbreviations: SD: standard deviation; GP: general practitioner; TSC: tuberous sclerosis complex; IGE: idiopathic generalized epilepsy; FE: focal epilepsy

Patients with TSC (mean: €169, median: €62, SD: €270) had significantly higher costs for diagnostic tests, such as magnetic resonance imaging, computed tomography, or X-ray imaging, than patients with IGE (mean: €67, median: €23, SD: €132; *P* = 0.001) and patients with FE (mean: €79, median: €32, SD: €118; *P* = 0.014). The highest diagnostic costs were associated with magnetic resonance imaging, electroencephalography, and blood sampling.

Additional costs for medical aids were significantly higher for patients with TSC (mean: €63, median: €0, SD: €254) than for patients with IGE (mean: €0.90, median: €0, SD: €6; *P* = 0.009) and FE (mean: €5, median: €0, SD: €45; *P* = 0.009). Among patients with TSC, 12% reported costs for medical aids, including compression hosiery (4%), helmets (3%), orthopedic aids (2%), and others. Only 2% of patients with IGE and FE reported costs for medical aids.

Care grade costs, which were not included in total direct costs, were significantly higher for patients with TSC (mean: €1257, median: €1635, SD: €1081) than for patients with IGE (mean: €21, median: €0, SD: €139; *P* < 0.001) and FE (mean: €184, median: €0, SD: €529; *P* < 0.001).

### Indirect (productivity) costs

Total indirect costs among working-age patients with TSC during the 3-month study period (mean: €7185, median: €11,925, SD: €5643) were significantly higher than among patients with IGE (mean: €3599, median: €0, SD: €5062; *P* < 0.001) and FE (mean: €5082, median: €2981, SD: €5284; *P* = 0.03).

The main contributor to indirect costs in the TSC cohort was the inability to work (*n* = 27; 29%), resulting in a mean cost of €3500 (median: €0, SD: €5460), which was significantly higher than the costs for patients with IGE (mean: €389, median: €0, SD: €2130; *P* < 0.001) and FE (mean: €648, median: €0, SD: €2718; *P* < 0.001). The largest contributor to indirect costs for the IGE cohort was early retirement (mean: €1037, median: €0, SD: €3379), whereas, in the FE cohort, the largest contributor was unemployment (mean: €1555, median: €0, SD: €4038), with no significant differences in these contributors among cohorts.

More patients with TSC were in disability workshops and caused significantly higher costs (mean: €1296, median: €0, SD: €3732) than patients with IGE (mean: €259, median: €0, SD €1749; *P* = 0.02) and FE (mean: €259, median: €0, SD: €1749; *P* = 0.02). The details of indirect costs can be found in Table 3.

**Table 3:** Total mean and median indirect (productivity) costs per disease cohort over three months measured in Euros (€)

	TSC (*N* = 92)		IGE (*N* = 92)		FE (*N* = 92)		*P*-value	*P*-value	*P*-value	95% CI ^(a)^		
	mean	median	mean	median	mean	median	TSC/IGE	TSC/FE	FE/IGE	TSC	IGE	FE
Indirect (productivity) costs												
Inability to work***†††	3499.7	0	388.9	0	648.1	0	< 0.001	< 0.001	1.00	2410.4–4671.6	0.0–816.2	138.7–1216.8
Reduction of working hours/part-time†	366.2	0	876.6	0	1017.5	0	0.54	0.04	0.74	125.7–640.2	432.2–1382.7	597.0–1478.0
Unemployment	1425.8	0	648.1	0.0	1555.4	0	0.43	1.00	0.27	701.5–2262.4	137.1–1257.4	833.0–2357.0
Early retirement	388.9	0	1037.0	0	907.3	0	0.41	0.70	1.00	0.00–813.1	425.9–1759.6	357.8–1495.7
Days off	207.9	0	129.6	0	176.0	0	1.00	1.00	1.00	69.0–370.5	47.8–231.9	72.2–299.2
Homemaker	0	0	259.2	0	518.5	0	0.94	0.13	0.94	N/A	0–643.5	128.2–1014.9
Pensions for disability workshops*†	1296.2	0	259.2	0	259.2	0	0.02	0.02	1.00	621.1–2125.2	0–620.0	0–608.4
Total indirect costs***†	7184.7	11,925	3598.5	0	5082.1	2981.3	< 0.001	0.03	0.11	6103.2–8317.1	2531.7–4674.3	4048.1–6160.5

*P < 0.05, **P < 0.01, and ***P < 0.001 between patients with TSC and IGE; †*P* < 0.05,††*P* < 0.01, and †††*P* < 0.001 between patients with TSC and FE

^a)^ 95% confidence intervals were calculated for costs using the bias-corrected accelerated (BCa) bootstrap approach

TSC: tuberous sclerosis complex; IGE: idiopathic generalized epilepsy; FE: focal epilepsy; CI: confidence interval

### Quality of life

**Fig. 3:**
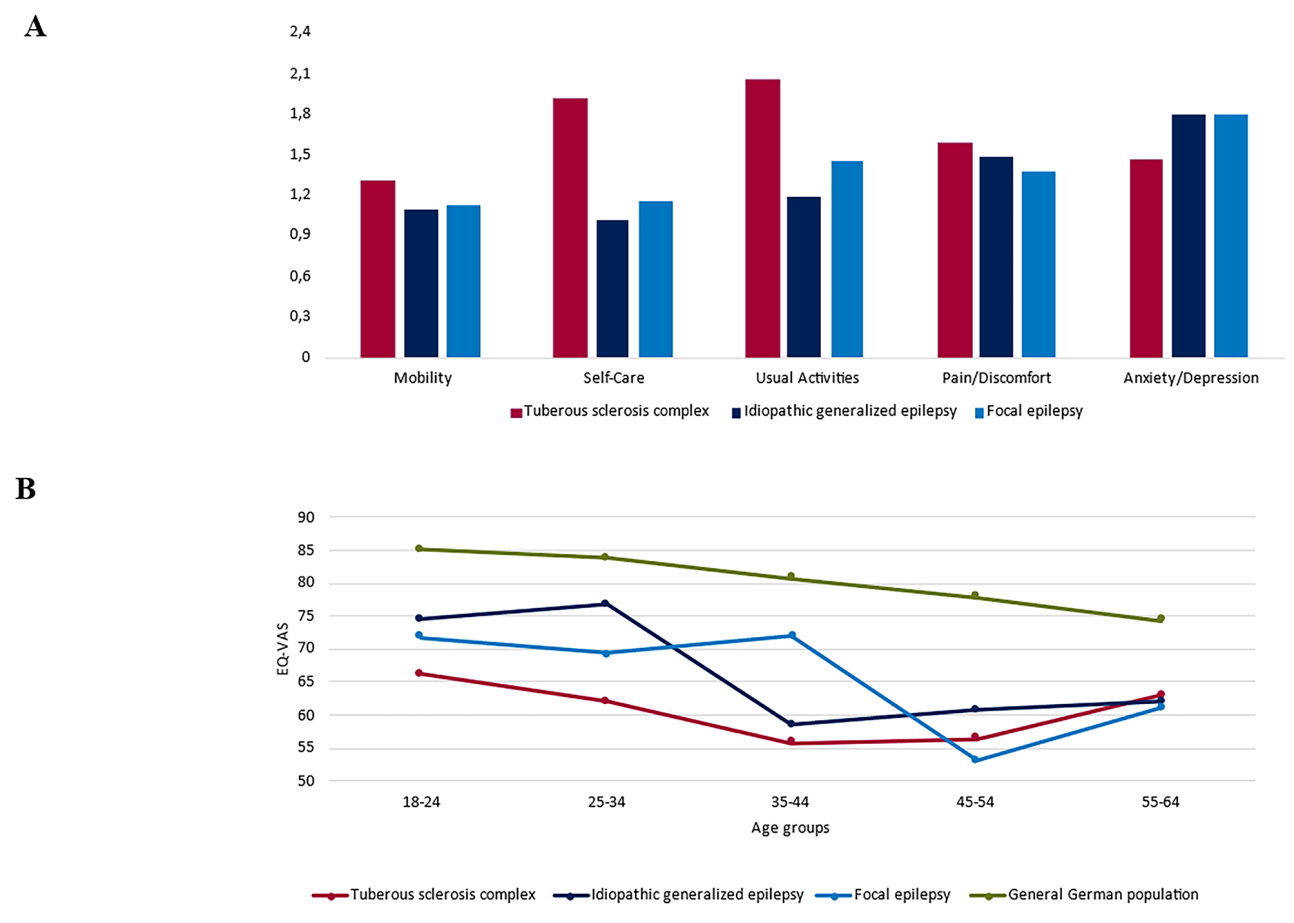
**A**. Mean EuroQuol-5-Dimension (EQ-5D) component scores by cohort **B**. Average self-rated EuroQuol Visual Analog Scale (EQ-VAS) score by age group in each disease cohort and general German population

Generic and health-related QoL were analyzed using the summary index scores from the EQ-5D-3L (TTO and VAS scores for Germany) and QOLIE-31 questionnaires. Patients with TSC had significantly lower EQ-5D-3L summary index scores (TTO, mean: 0.705, SD: 0.314; VAS, mean: 0.577, SD: 0.318) than patients with IGE (TTO, mean: 0.897, SD: 0.184; VAS, mean: 0.813, SD: 0.199; *P* < 0.001) or FE (TTO, mean: 0.879, SD 0.194; VAS mean 0.769, SD 0.227; *P* < 0.001; Table 4). Additionally self-rated mean VAS scores were statistically significantly lower in patients with TSC (61.8) than in patients with IGE (70.7; *P* = 0.004) and FE (68.8; *P* = 0.045). Mean self-rated VAS scores by age-groups are presented in Fig. 3B. Patients with TSC most frequently reported difficulties for ‘usual activities’ (67%, mean component score: 2.05), ‘self-care’ (54%, mean component score: 1.91), and ‘pain/discomfort’ (49%, mean component score: 1.58), whereas patients with IGE and FE most frequently reported difficulties with ‘anxiety and depression’ (IGE: 74%, mean component score: 1.80; FE: 66%, mean component score: 1.79; Fig. 3A).

**Table 4:** Scores of various quality-of-life, symptom of depression, stigma, seizure worry and adverse events surveys among the three cohorts

		TSC (*N* = 92)		IGE (*N* = 92)						FE (*N* = 92)			General population	*P*-value TSC vs. IGE	*P*-value TSC vs. FE	*P*-value IGE vs. FE
		mean	N (%)	Mean	N (%)					mean	N (%)					
EQ-5D-3L																
	EQ-5D-3L (TTO) ***†††	0.705		0.897						0.879			0.915	< 0.001	< 0.001	1
	EQ-5D-3L (VAS) ***†††	0.577		0.813						0.769			0.890	< 0.001	< 0.001	0.7
	EQ-5D-3L (VAS self-rated) **†	61.8		70.7						68.8			78.3	0.004	0.045	1
QOLIE-31													-			
	Seizure worry †	67.71		67.06						58.62				1	0.042	0.2
	Overall quality of life *	61.72		67.53						62.74				0.046	1	0.14
	Emotional well-being	62.01		65.14						61.36				0.7	1	0.5
	Energy/Fatigue	47.42		55.11						47.57				1	1	1
	Cognitive function **	51.16		66.24						53.27				0.003	1	0.007
	Medication effects	66.27		64.83						56.49				1	0.1	0.1
	Social function***	56.95		76.77						61.69				< 0.001	1	< 0.001
	Overall scores**	57.72		66.55						57.64				0.004	1	0.004
NDDI-E**		13.10		11.15						12.04			-	0.009	0.2	0.6
	No symptoms of depression (< 14)		41 (44.6)			70 (76.1)					56 (60.9)			0.2^a^	1.0^a^	–
	Symptoms of depression (≥ 14)		26 (28.3)			21 (22.8)					34 (37.0)					
	Incomplete		25 (27.2)			1 (1.1)					2 (2.2)					
rESS (Jacoby Stigma Scale) ***†††		3.97		1.48						2.45			-	< 0.001	< 0.001	0.027
	No stigma (0)		6 (6.5)			49 (53.3)					35 (38.0)					
	Mild or moderate stigma (1–6)		54 (58.7)			37 (40.2)					47 (51.1)			< 0.001^a^	< 0.001^a^	-
	High stigma (7–9)		13 (14.1)			5 (5.4)					10 (10.9)					
	Incomplete		19 (20.7)			1 (1.1)					0 (0)					
Seizure Worry Scale		3.28		3.17						3.75			-	1	0.4	0.2
	No or mild seizure worry (0–2)		24 (26.1)			33 (35.9)					23 (25.0)					
	Moderate or high worry (3–6)		40 (43.5)			59 (64.1)					68 (73.9)			1^a^	0.6^a^	-
	Incomplete		28 (30.4)			0 (0)					1 (1.1)					
LAEP*		42.74		37.47						40.21			-	0.017	0.6	0.4
	No relevant adverse events (< 35)		20 (21.7)			37 (40.2)					30 (32.6)			0.06^a^	0.8^a^	-
	Relevant adverse events (≥ 35)		69 (75.0)			55 (59.8)					62 (67.4)					
	Incomplete		3 (3.2)			0 (0)					0 (0)					

*P < 0.05, **P < 0.01, and ***P < 0.001 between patients with TSC and patients with IGE; †*P* < 0.05, ††*P* < 0.01, and †††*P* < 0.001 between patients with TSC and patients with FE

^(a)^ Chi-Square tests followed by Bonferroni correction were used to test the differences between the proportions of patients with TSC and the proportions of patients with IGE and FE

Due to rounding, numbers may not add up to 100%. Abbreviations: EQ-5D-3L: EuroQol scale with 5 dimensions and 3 levels; QOLIE-31: The Quality of Life in Epilepsy Inventory with 31 items; NDDI-E: Neurological Disorder Depression Inventory for Epilepsy; rESS: revised version of the Epilepsy Stigma Scale; LAEP: Liverpool Adverse Events Profile; TTO: time-trade-off; VAS: visual analog scale; TSC: tuberous sclerosis complex; IGE: idiopathic generalized epilepsy; FE: focal epilepsy

The QOLIE-31 overall scores were significantly lower for the TSC (57.7) and FE (57.6) cohorts than for the IGE cohort (66.6; *P* = 0.004 for both comparisons). Patients with TSC and FE had lower subscale scores for ‘cognitive function’ (*P* = 0.003 and *P* = 0.007, respectively) and ‘social function’ (*P* < 0.001 for both comparisons) than patients with IGE. Patients with TSC had significantly lower subscale scores for ‘overall QoL’ than patients with IGE (*P* = 0.046), while there was no significantly difference between FE patients and both other cohorts (Table 4).

Depressive symptoms were analyzed using data collected from the NDDI-E, revealing a significant difference (*P* = 0.009) between patients with TSC (mean: 13.1, SD: 4.28) and patients with IGE (mean: 11.15, SD: 4.07). No differences in the proportions of patients reaching the cutoff score of 14 (indicating depressive mood) were observed among the three cohorts.

To assess epilepsy-related stigma, we examined rESS responses. Higher rESS scores were observed in the TSC cohort (mean: 3.97, SD: 2.69) than in the other cohorts (IGE, mean: 1.48, SD: 2.12; *P* < 0.001; FE, mean: 2.45, SD: 2.57; *P* < 0.001). A higher proportion of patients with TSC (67/92) experienced moderate or high stigma than patients with IGE (42/92) or FE (57/92; *P* < 0.001, Table 4).

Seizure Worry Scale scores were similar across the three cohorts, with mean scores of 3.28 (SD 1.90) among patients with TSC, 3.17 (SD 1.91) among patients with IGE, and 3.75 (SD 1.80) among patients with FE.

To analyze therapy-related adverse events, LAEP scores were obtained. Patients with TSC had significantly higher LAEP scores (mean: 42.74, SD: 10.60) than patients with IGE (mean: 37.47, SD: 11.35; *P* = 0.017), while patients with FE (mean: 40.21, SD: 12.46) showed no significantly difference. When we dichotomized LAEP scores (LAEP scores ≥ 35 vs. <35), no significant differences were observed in the proportions of each cohort with and without therapy-related adverse events. In all three cohorts, fatigue and problems with concentration were the most frequently reported therapy-related adverse events, followed by restlessness and memory problems.

## Discussion

Previous studies have compared the economic burden of disease and QoL between patients with TSC suffering from various organ manifestations (e.g., epilepsy) and epilepsy patients without TSC. However, to our knowledge, no comparative studies in Germany have specifically examined the costs of illness and QoL in patients with TSC related epilepsy and patients with explicit epilepsy types, such as IGE and FE.

Our analysis indicates that more patients with TSC are in possession of disability ID and that these patients receive higher care levels and incur significantly higher care level costs than patients with IGE and FE. This fact suggests that TSC results in marked limitations on the daily lives of patients with TSC compared to patients with IGE and FE, which is consistent with previous studies on costs and burden on caregivers [7, 41–44].

Our analysis showed higher total direct costs for patients with TSC than for patients with IGE and FE. Mean direct costs for patients with TSC were twice as high as those for patients with IGE and FE due to different components, such as the use of mTOR inhibitors [45]. Zöllner et al. [17] identified the use of expensive mTOR inhibitors as an independent cost-driving factor for patients with TSC. Betts et al. [46] reported that mTOR inhibitors were a key cost component for patients with TSC in the US compared with patients with other epilepsy syndromes. However, mTOR inhibitor prices are likely to decrease as generic formulations become available, which may result in a convergence of the drug treatment costs borne by the TSC cohort and the other cohorts [47, 48].

In this analysis, no significant differences in the number of ASMs or associated costs were identified among the three cohorts, patients with FE have a higher ASM drug load measured by DDD. Patients with TSC used a mean of 1.7 ASMs compared with 1.5 for patients with IGE and 1.9 for patients with FE. In the comparative study reported by Betts et al., patients with TSC used a mean of 2.1 ASMs, which was significantly higher than the mean of 1.3 ASMs reported for the epilepsy cohort without TSC [46]. However, 58% of patients with TSC in our study received at least 2 ASMs, which is consistent with results from Shepherd et al., in which 60% of patients with TSC diagnosed with epilepsy used at least 2 ASMs [49].

A significant difference in direct costs due to other prescribed drugs was observed in this analysis, as patients with TSC reported costs of €144 compared with €31 for patients with IGE and €34 for patients with FE. Despite ensuring that the cohorts were matched for age and sex, patients with TSC report significantly higher costs for cardiovascular/antithrombotic agents and alimentary tract/metabolic drugs than patients with IGE and FE, particularly drugs associated with lipid and glucose metabolism and blood pressure regulation. Previous studies, such as the Dutch study reported by Mulder et al. [50], identified hypercholesterolemia as a frequent side effect of mTOR inhibitors. In the present analysis, 11 of 31 patients with TSC using mTOR inhibitors also reported at least one metabolic or cardiovascular drug associated with a mean cost of €41.

Major contributors to direct costs included outpatient visits with healthcare professionals, outpatient hospital visits, diagnostic procedures, and ancillary treatments. For most of these categories, the costs for patients with TSC were at least twice as high as those for patients with IGE and FE, which may be related to the multisystem nature of TSC. Multiple organ manifestations likely require continuous monitoring and additional diagnostic procedures and treatments, increasing the overall economic burden [49] compared with IGE and FE.

In the present study, indirect costs due to a loss of productivity were significantly higher for patients with TSC (mean €7185) than for patients with IGE (€3599) and FE (€5082). Only 40% of patients with TSC were employed (full-time, part-time, reduced hours, or vocational training), whereas 77% of patients with IGE and 66% of patients with FE were employed. A comparison between the general German population and patients with IGE shows an equal number of employed individuals, whereas patients with TSC are almost half as likely to be employed than individuals from the general German population [51] (Fig. 1A). In a US-based study, Skalicky et al. [52] reported that 70% of participating patients with TSC were employed; however, 58% rated their work time as impaired due to TSC. The different social systems between Germany and the US might explain the higher number of patients with TSC who reported employment in the US compared with our study. Among patients with FE in our study, 34% were not employed. Ioannou et al. [53] reported that 46% of patients with FE were not employed, which differs from our results; however, the patients in the study by Ioannou et al. were all defined as drug refractory, which was not the case in our study.

Based on generic QoL scores obtained from the EQ-5D-3L questionnaire, patients with TSC showed significantly lower index scores with TTO and VAS methods than the other cohorts in the present study. With a mean TTO score of 0.705 and a VAS score of 0.577, patients with TSC experienced statistically significantly considerably worse generic QoL than the overall German population (*P* < 0.001). Janssen et al. [54] reported a pooled mean population-weighted TTO value of 0.915 and VAS value of 0.890 for the five largest European economies, including Germany, indicating a mean reduction in TTO of 0.210 and in VAS of 0.313 for patients with TSC in our study relative to the general German population, indicating reduced generic QoL. EQ-5D-3L values for patients with IGE (TTO, 0.897; VAS, 0.813) and FE (TTO, 0.879; VAS, 0.769) were also lower than those for the general German population; however only the reductions of VAS values were statistically significant (*P* < 0.001 in both comparisons). Additionally self-rated overall mean VAS scores were significantly lower in all cohorts (TSC:61.8; IGE:70.7; FE:68.8) compared to the German population (78.3). When dividing the cohort in different age-groups, all 3 cohorts presented statistically significantly lower self-rated VAS scores than the general German population except of age-group 55–64 (Fig. 3B).

The EQ-5D-3L subscores revealed that patients with TSC frequently reported difficulties with usual activities (67%), self-care (54%), and pain/discomfort (49%). In the TOSCA study, Jansen et al. [41] found that patients with TSC reported difficulties with pain/discomfort (37%) and anxiety/depression (47%). However, the TOSCA study allowed caregivers to complete the EQ-5D-3L for patients who could not complete the questionnaire independently, resulting in increased overall impairment, which likely had direct consequences on generic QoL outcomes. Our results were limited to self-reported information, which may indicate that the overall effects of TSC on general QoL and EQ-5D-3L subscores may be underestimated by our study.

Differences in QOLIE-31 overall scores were identified between patients with TSC and IGE, whereas patients with FE reported similar overall scores as patients with TSC. The mean social and cognitive function subscores were significantly lower for patients with TSC (social, 56.95; cognitive, 51.16) than for patients with IGE (social, 76.77; cognitive, 66.24). The TOSCA study used the modified QOLIE-31-P questionnaire [41], which includes an additional question regarding distress and worries related to epilepsy. The overall QOLIE-31-P scores in the TOSCA study were comparable to our results, although the mean cognition scores of the TOSCA study were more than 10 points higher (mean score 63.6) [41] for patients with TSC than in our study. However, only 24 individuals from the TOSCA study completed the questionnaire.

The rESS scores revealed that patients with TSC experienced significantly increased epilepsy-related stigma (3.97) than patients with IGE (1.48) and FE (2.45). A larger proportion of patients with TSC reported moderate or severe stigma than those with IGE and FE. In a Croatian study by Bielen et al. [37], 53% of 298 investigated epilepsy patients reported feelings of stigma, with 45% experiencing mild to moderate stigma and 8% reporting severe stigma. These results were consistent for patients with IGE and FE in our study, but our study had slightly higher proportions for patients with TSC (59% mild to moderate stigma and 14% high stigma). Zöllner et al. [16] identified an association between the range of clinical TSC manifestations and severe stigma, as measured by the Epilepsy Stigma Scale.

### Limitations

Although our comparative analysis was based on studies that used similar questionnaires, with question-by-question matching, slight wording differences could have affected how each question was perceived by respondents. In addition, the studies were performed in two consecutive years (2019 and 2020), and in the intervening time, healthcare and treatment guidelines may have changed. However, we attempted to adjust both direct and indirect costs to improve comparability.

Because we limited our study to patients able to provide independent informed consent, our study excluded the most severely affected patients, which likely resulted in the underestimation of the burden of illness and true QoL. Patients with TSC also tended not to respond to all components of the QoL questionnaires, which further limits our assessment.

Although the present study highlights the costs and QoL burden associated with TSC, IGE, and FE, this was not an interventional study; therefore, we are unable to draw any conclusions regarding how to ease the disease burden experienced by patients and caregivers.

To facilitate a more comprehensive comparison of the three groups, additional information on the specific comorbidities present in each group, the cognitive levels of the patients, and the underlying pathologies of focal epilepsies would have been beneficial. Moreover, considering the variable severity of TSC, it would have been insightful to explore whether epilepsy or comorbidities predominantly influenced the degree of disability and associated burden. However, this information was not surveyed in detail, which represents a limitation.

## Conclusions

The high QoL and monetary burdens associated with TSC are apparent, reinforcing existing research into preventive treatments [55, 56]. Patients with TSC and epilepsy generally experience higher burdens than patients with IGE and FE. This study supports the observation that TSC is a multisystem disorder with epilepsy that severely limits the daily lives of patients, resulting in high economic costs. Further research and efforts should focus on identifying the drivers of high healthcare resource use and opportunities to decrease monetary impacts and improve QoL among patients with TSC.

### Electronic Supplementary Material

Below is the link to the electronic supplementary material.

**Supplementary Material**: **Additional File 1** Demographic factors and clinical characteristics of cohorts; **Additional File 2** Reported frequency of seizures; **Additional File 3** Number of patients per cohort taking the indicated prescribed drugs according to the Anatomical Therapeutic Chemical (ATC) classification system excluding ASMs (ATC N03A) and mTOR inhibitors (ATC L01EG)

## Data Availability

The datasets used and analyzed during the current study are available from the corresponding author on reasonable request.

## References

[1] Ebrahimi-Fakhari D, Mann LL, Poryo M, Graf N, von Kries R, Heinrich B (2018). Incidence of tuberous sclerosis and age at first diagnosis: New data and emerging trends from a national, prospective surveillance study. Orphanet Journal of Rare Diseases.

[2] Nabbout R, Belousova E, Benedik MP, Carter T, Cottin V, Curatolo P (2019). Epilepsy in tuberous sclerosis complex: Findings from the TOSCA Study. Epilepsia Open.

[3] Rosset, C., Netto, C. B. O., & Ashton-Prolla, P. (2017). TSC1 and TSC2 gene mutations and their implications for treatment in tuberous sclerosis complex: A review. *Genetics and Molecular Biology*, *40*(1), 69–79. 10.1590/1678-4685-GMB-2015-0321 10.1590/1678-4685-GMB-2015-0321 PMC5409767 28222202

[4] Dorn, T. (2022). Tuberous sclerosis complex (TSC). *Zeitschrift für Epileptologie*, *35*(3), 242–249. 10.1007/s10309-022-00512-w

[5] Chu-Shore CJ, Major P, Camposano S, Muzykewicz D, Thiele EA (2010). The natural history of epilepsy in tuberous sclerosis complex. Epilepsia.

[6] Curatolo P, Moavero R, de Vries PJ (2015). Neurological and neuropsychiatric aspects of tuberous sclerosis complex. Lancet Neurology.

[7] Zollner JP, Franz DN, Hertzberg C, Nabbout R, Rosenow F, Sauter M (2020). A systematic review on the burden of illness in individuals with tuberous sclerosis complex (TSC). Orphanet Journal of rare Diseases.

[8] Specchio N, Nabbout R, Aronica E, Auvin S, Benvenuto A, de Palma L (2023). Updated clinical recommendations for the management of tuberous sclerosis complex associated epilepsy. European Journal of Paediatric Neurology: EJPN : Official Journal of the European Paediatric Neurology Society.

[9] Strzelczyk, A., Kurlemann, G., Bast, T., Bettendorf, U., Kluger, G., Mayer, T., et al. (2022). Exploring the relationships between composite scores of disease severity seizure-freedom and quality of life in Dravet syndrome. *Neurological Research and Practice*, *4*(1), 22. 10.1186/s42466-022-00186-9 10.1186/s42466-022-00186-9 PMC9169336 35659154

[10] Willems LM, Hochbaum M, Zollner JP, Schulz J, Menzler K, Langenbruch L (2022). Trends in resource utilization and cost of illness in patients with active epilepsy in Germany from 2003 to 2020. Epilepsia.

[11] Willems LM, Hochbaum M, Frey K, Schulz J, Menzler K, Langenbruch L (2022). Multicenter, cross-sectional study of the costs of illness and cost-driving factors in adult patients with epilepsy. Epilepsia.

[12] Strzelczyk A, Reese JP, Dodel R, Hamer HM (2008). Cost of epilepsy: A systematic review. Pharmacoeconomics.

[13] Kingswood JC, Crawford P, Johnson SR, Sampson JR, Shepherd C, Demuth D (2016). The economic burden of tuberous sclerosis complex in the UK: A retrospective cohort study in the Clinical Practice Research Datalink. Journal of Medical Economics.

[14] Kingswood JC, Nasuti P, Patel K, Myland M, Siva V, Gray E (2016). The economic burden of tuberous sclerosis complex in UK patients with renal manifestations: A retrospective cohort study in the clinical practice research datalink (CPRD). Journal of Medical Economics.

[15] Grau J, Zollner JP, Schubert-Bast S, Kurlemann G, Hertzberg C, Wiemer-Kruel A (2021). Direct and indirect costs and cost-driving factors of tuberous sclerosis complex in children, adolescents, and caregivers: A multicenter cohort study. Orphanet Journal of rare Diseases.

[16] Zollner JP, Conradi N, Sauter M, Knuf M, Knake S, Kurlemann G (2021). Quality of life and its predictors in adults with tuberous sclerosis complex (TSC): A multicentre cohort study from Germany. Neurol Res Pract.

[17] Zollner JP, Grau J, Rosenow F, Sauter M, Knuf M, Kurlemann G (2021). Direct and indirect costs and cost-driving factors in adults with tuberous sclerosis complex: A multicenter cohort study and a review of the literature. Orphanet Journal of Rare Diseases.

[18] Korbel K, Rosenow F, Maltseva M, Muller H, Schulz J, Tsalouchidou PE (2022). Impact of COVID-19 pandemic on physical and mental health status and care of adults with epilepsy in Germany. Neurol Res Pract.

[19] Hochbaum M, Kienitz R, Rosenow F, Schulz J, Habermehl L, Langenbruch L (2022). Trends in antiseizure medication prescription patterns among all adults, women, and older adults with epilepsy: A German longitudinal analysis from 2008 to 2020. Epilepsy & Behavior.

[20] Siebenbrodt K, Willems LM, von Podewils F, Mross PM, Struber M, Langenbruch L (2023). Determinants of quality of life in adults with epilepsy: A multicenter, cross-sectional study from Germany. Neurol Res Pract.

[21] Willems LM, Zollner JP, Hamann L, Knake S, Kovac S, von Podewils F (2023). Unemployment and early retirement among patients with epilepsy - A study on predictors, resilience factors and occupational reintegration measures. Epilepsy & Behavior.

[22] Willems LM, van der Goten M, von Podewils F, Knake S, Kovac S, Zollner JP (2023). Adverse event profiles of antiseizure medications and the impact of Coadministration on Drug Tolerability in adults with Epilepsy. CNS Drugs.

[23] Scheffer IE, Berkovic S, Capovilla G, Connolly MB, French J, Guilhoto L (2017). ILAE classification of the epilepsies: Position paper of the ILAE Commission for Classification and terminology. Epilepsia.

[24] Fisher RS, Cross JH, French JA, Higurashi N, Hirsch E, Jansen FE (2017). Operational classification of seizure types by the International League against Epilepsy: Position paper of the ILAE Commission for classification and terminology. Epilepsia.

[25] Northrup H, Krueger (2013). Tuberous sclerosis complex diagnostic criteria update: Recommendations of the 2012 Iinternational Tuberous Sclerosis Complex Consensus Conference. Pediatric Neurology.

[26] von Elm E, Altman DG, Egger M, Pocock SJ, Gotzsche PC, Vandenbroucke JP (2007). The strengthening the reporting of Observational studies in Epidemiology (STROBE) statement: Guidelines for reporting observational studies. Lancet.

[27] Bock JO, Brettschneider C, Seidl H, Bowles D, Holle R, Greiner W (2015).

[28] Schwendler M, Flessa S (2019). [The calculated physician’s fee in the Doctor’s fee scale (EBM): A normative parameter for medical doctors in the Outpatient Sector]. Gesundheitswesen (Bundesverband Der Arzte Des Offentlichen Gesundheitsdienstes (Germany)).

[29] Schwabe, U., & Ludwig, W. D. (2020). *Arzneiverordnungs-Report 2020*. Springer Nature.

[30] Hersi H, Raitanen J, Saarinen JT, Peltola J (2023). Prescribed antiseizure medication doses and their relation to defined daily doses for achieving seizure freedom in newly diagnosed patients with epilepsy. Epilepsia Open.

[31] EuroQol Group (1990). EuroQol - a new facility for the measurement of health-related quality of life. Health Policy (Amsterdam Netherlands).

[32] Cramer JA, Perrine K, Devinsky O, Bryant-Comstock L, Meador K, Hermann B (1998). Development and cross-cultural translations of a 31-item quality of life in epilepsy inventory. Epilepsia.

[33] Gilliam FG, Barry JJ, Hermann BP, Meador KJ, Vahle V, Kanner AM (2006). Rapid detection of major depression in epilepsy: A multicentre study. Lancet Neurology.

[34] Strzelczyk A, Aledo-Serrano A, Coppola A, Didelot A, Bates E, Sainz-Fuertes R (2023). The impact of epilepsy on quality of life: Findings from a European survey. Epilepsy &amp; Behavior: E&amp;B.

[35] Romoli M, Eusebi P, Siliquini S, Bedetti C, Calabresi P, Costa C (2018). Liverpool adverse events Profile: Italian validation and predictive value for dropout from antiepileptic treatment in people with epilepsy. Epilepsy & Behavior.

[36] Baker GA, Brooks J, Buck D, Jacoby A (2000). The stigma of epilepsy: A European perspective. Epilepsia.

[37] Bielen I, Friedrich L, Sruk A, Prvan MP, Hajnsek S, Petelin Z (2014). Factors associated with perceived stigma of epilepsy in Croatia: A study using the revised Epilepsy Stigma Scale. Seizure.

[38] Jacoby A, Lane S, Marson A, Baker GA (2011). Relationship of clinical and quality of life trajectories following the onset of seizures: Findings from the UK MESS Study. Epilepsia.

[39] Desgagne A, Castilloux AM, Angers JF, LeLorier J (1998). The use of the bootstrap statistical method for the pharmacoeconomic cost analysis of skewed data. Pharmacoeconomics.

[40] Barber JA, Thompson SG (2000). Analysis of cost data in randomized trials: An application of the non-parametric bootstrap. Statistics in Medicine.

[41] Jansen AC, Vanclooster S, de Vries PJ, Fladrowski C, Beaure d’Augeres G, Carter T (2020). Burden of illness and quality of life in Tuberous Sclerosis Complex: Findings from the TOSCA Study. Frontiers in Neurology.

[42] Fagnani F, Laurendeau C, de Zelicourt M, Marshall J (2022). Epidemiology and disease burden of tuberous sclerosis complex in France: A population-based study based on national health insurance data. Epilepsia Open.

[43] Chu WC, Chiang LL, Chan DC, Wong WH, Chan GC (2020). Prevalence, mortality and healthcare economic burden of tuberous sclerosis in Hong Kong: A population-based retrospective cohort study (1995–2018). Orphanet Journal of rare Diseases.

[44] Skrobanski H, Vyas K, Bowditch S, Hubig L, Dziadulewicz E, Fish L (2023). The Burden of Caring for individuals with Tuberous Sclerosis Complex (TSC) who experience epileptic seizures: A descriptive UK Survey. Pharmacoecon Open.

[45] Kerling, F. (2022). New developments in anticonvulsive therapy for people with intellectual disability. *Zeitschrift für Epileptologie*, *35*(3), 225–229. 10.1007/s10309-022-00509-5

[46] Betts KA, Stockl KM, Yin L, Hollenack K, Wang MJ, Yang X (2020). Economic burden associated with tuberous sclerosis complex in patients with epilepsy. Epilepsy & Behavior.

[47] Willems LM, Rosenow F, Schubert-Bast S, Kurlemann G, Zollner JP, Bast T (2021). Efficacy, Retention and Tolerability of Everolimus in patients with tuberous sclerosis complex: A Survey-based study on patients’ perspectives. Cns Drugs.

[48] Willems, L. M., Strzelczyk, A., & Rosenow, F. (2021). Everolimus as disease-specific treatment option in tuberous sclerosis complex-associated drug refractory epilepsy - a systematic review. *Zeitschrift für Epileptologie*, *34*, 168–174. 10.1007/s10309-020-00393-x

[49] Shepherd C, Koepp M, Myland M, Patel K, Miglio C, Siva V (2017). Understanding the health economic burden of patients with tuberous sclerosis complex (TSC) with epilepsy: A retrospective cohort study in the UK Clinical Practice Research Datalink (CPRD). BMJ open.

[50] Mulder FVM, Peeters E, Westerink J, Zwartkruis FJT, de Ranitz-Greven WL (2022). The long-term effect of mTOR inhibition on lipid and glucose metabolism in tuberous sclerosis complex: Data from the Dutch TSC registry. Orphanet Journal of rare Diseases.

[51] Bundeszentrale für politische Bildung. Erwerbstätigenquote nach Geschlecht und Alter. Available from https://www.bpb.de/kurz-knapp/zahlen-und-fakten/soziale-situation-in-deutschland/61688/erwerbstaetigenquoten-nach-geschlecht-und-alter/ [accessed 2023].

[52] Skalicky AM, Rentz AM, Liu Z, Said Q, Nakagawa JA, Frost MD (2018). Economic burden, work, and school productivity in individuals with tuberous sclerosis and their families. Journal of Medical Economics.

[53] Ioannou P, Foster DL, Sander JW, Dupont S, Gil-Nagel A, Drogon O’Flaherty E (2022). The burden of epilepsy and unmet need in people with focal seizures. Brain Behav.

[54] Janssen MF, Pickard AS, Shaw JW (2021). General population normative data for the EQ-5D-3L in the five largest European economies. The European Journal of Health Economics.

[55] Kotulska K, Kwiatkowski DJ, Curatolo P, Weschke B, Riney K, Jansen F (2021). Prevention of Epilepsy in infants with Tuberous Sclerosis Complex in the EPISTOP Trial. Annals of Neurology.

[56] Huschner F, Glowacka-Walas J, Mills JD, Klonowska K, Lasseter K, Asara JM (2023). Molecular EPISTOP, a comprehensive multi-omic analysis of blood from tuberous sclerosis complex infants age birth to two years. Nature Communications.

