# Supplementary material for: A multicenter, matched case–control analysis comparing burden of illness among patients with tuberous sclerosis complex related epilepsy, generalized idiopathic epilepsy, and focal epilepsy in Germany
Source: Neurol Res Pract. 2024 May 30;6:29. doi: 10.1186/s42466-024-00323-6 (PMC11138101; doi:10.1186/s42466-024-00323-6)
Supplement: Supplementary file 1 — Supplementary Material: Additional File 1 Demographic factors and clinical characteristics of cohorts; Additional File 2 Reported frequency of seizures; Additional File 3 Number of patients per cohort taking the indicated prescribed drugs according to the Anatomical Therapeutic Chemical (ATC) classification system excluding ASMs (ATC N03A) and mTOR inhibitors (ATC L01EG) [file 42466_2024_323_MOESM1_ESM.pdf]

Electronic Supplementary Material

Additional File 1: Demographic factors and clinical characteristics of cohorts

		TSC			IGE			FE			P-value	P-value	P-value
Demographics and clinical characteristics		Mean	Median	N (%)	Mean	Median	N (%)	Mean	Median	N (%)	TSC vs. IGE	TSC vs. FE	FE vs. IGE
Age (years)		32.0	29.0		31.8	30.0		32.0	29.5		1.00	1.00	1.00
Age (years) at first epileptic symptoms***†††		1.9	0		16.9	16		15.9	14		<0.001	<0.001	0.56
Patients' missed workdays		1.6	0		1.0	0		1.4	0		1.00	1.00	1.00
Sex	Female			60 (65.2)			60 (65.2)			60 (65.2)	1.00[a]	1.00[a]	-
	Male			32 (34.8)			32 (34.8)			32 (34.8)	1.00[a]	1.00[a]	-
Patients' employment status***†††				34 (40.0)			71 (77.2)			59 (65.6)	<0.001[a]	<0.001[a]	-
	Missing			7 (7.6)			0 (0)			2 (2.2)			
Proportion of patients with a disability card ***†††				84 (91.3)			34 (37.0)			53 (57.6)	<0.001[a]	<0.001[a]	-
Proportion of patients with a care grade***†††				62 (67.4)			4 (4.3)			13 (14.1)	<0.001[a]	<0.001[a]	-

	Missing			0 (0)			1 (1.0)			3 (3.3)			

[a]Chi-square tests were used to test hypotheses, followed by Bonferroni correction, regarding differences in the proportions of patients with TSC and the proportions of patients with IGE and FE.

*P<0.05, **P<0.01, and ***P<0.001 between patients with TSC and IGE; †P<0.05,††P<0.01, and †††P<0.001 between patients with TSC and FE.

TSC, tuberous sclerosis complex; IGE, idiopathic generalized epilepsy; FE, focal epilepsy

Additional File 2: Reported frequency of seizures

	TSC N (% of cohort)	IGE N (% of cohort)	FE N (% of cohort)	TSC/IGE P-value	TSC/FE P-value
At least once a day	17 (19)	6 (7)	1 (1)	<0.001[a]	1.00[a]
At least once a week	16 (17)	6 (7)	13 (14)		
At least once a month	13 (14)	9 (10)	25 (27)		
At least once every 6 months	6 (7)	8 (9)	9 (10)		
At least once a year	2 (2)	13 (14)	11 (12)		
No seizures for over a year	35 (38)	42 (46)	29 (32)		
Missing	3 (3)	8 (9)	4 (4)		
Total	92	92	92		

Due to rounding, numbers may not add up to 100%.

[a]Chi-square tests were used to compare hypotheses, followed by Bonferroni correction, regarding differences in the proportions of patients with TSC compared with the proportions of patients with IGE and FE who experience seizures daily, weekly, monthly vs. every six months, or less often.

TSC, tuberous sclerosis complex; IGE, idiopathic generalized epilepsy; FE, focal epilepsy.

Additional File 3: Number of patients per cohort taking the indicated prescribed drugs according to the Anatomical Therapeutic Chemical (ATC) classification system excluding ASMs (ATC N03A) and mTOR inhibitors (ATC L01EG)

			TSC N (%)	IGE N (%)	FE N (%)	P-value TSC vs. IGE[a]	P-value TSC vs. FE[a]
Alimentary tract and metabolism (ATC A)* †			14 (15.2)	2 (2.2)	3 (3.3)	0.012	0.03
		Drugs used in diabetes	2 (2.2)	0 (0)	0 (0)	0.93	0.93
		Drugs for acid related disorders	9 (9.8)	2 (2.2)	3 (3.3)	0.18	0.438
		Other	4 (4.3)	0 (0)	0 (0)	0.258	0.258
Cardiovascular system and antithrombotic agents (ATC C and B)**			23 (25)	7 (7.6)	11 (11.9)	0.006	0.138
	Antihypertensives*†		20 (21.7)	6 (6.5)	7 (7.6)	0.018	0.042
		Agents acting on the renin-angiotensin-system	10 (10.9)	4 (4.3)	3 (3.3)	0.57	0.264
		Beta blocking agents	7 (7.6)	3 (3.3)	3 (3.3)	1.00	1.00
		Calcium channel blockers	6 (6.5)	1 (1.1)	1 (1.1)	0.324	0.324
		Diuretics	5 (5.4)	1 (1.1)	0 (0)	0.582	0.138
	Lipid modifying agents		3 (3.3)	3 (3.3)	3 (3.3)	1.00	1.00

	Antithrombotic agents		2 (2.2)	2 (2.2)	1 (1.1)	1.00	1.00
	Other		1 (1.1)	0 (0)	0 (0)	1.00	1.00
Genito urinary system and sex hormones (ATC G)			6 (6.5)	1 (1.1)	2 (2.2)	0.324	0.888
	Sex hormones and modulators of the genital system		4 (4.3)	1 (1.1)	1 (1.1)	1.00	1.00
	Urologicals		2 (2.2)	0 (0)	1 (1.1)	0.93	0.93
Systemic hormonal preparations, excl. sex hormones and insulins (ATC H)			12 (13.0)	4 (4.3)	6 (6.5)	0.216	0.822
Antineoplastic and immunmodulating agents (excl. m-TOR; ATC L)			1 (1.1)	0 (0)	0 (0)	1.00	1.00
Musculo-skeletal system (ATC M)			1 (1.1)	0 (0)	0 (0)	1.00	1.00
Nervous system (ATC N)*** †††			32 (34.7)	9 (9.8)	9 (9.8)	<0.001	<0.001
	Psycholeptics***†††		21 (22.8)	2 (2.2)	4 (4.3)	<0.001	<0.001
		Antipsychotics, atypical***†	13 (14.1)	1 (1.1)	2 (2.2)	<0.001	0.018
		Antipsychotics, typical; high-potency	6 (6.5)	0 (0)	0 (0)	0.078	0.078

		Antipsychotics, typical; mid-potency	2 (2.2)	0 (0)	0 (0)	0.93	0.93
		Antipsychotics, typical; low-potency	5 (5.4)	0 (0)	0 (0)	0.138	0.138
		Other	1 (1.1)	2 (2.2)	2 (2.2)	1.00	1.00
	Psychoanaleptics		14 (15.2)	7 (7.6)	5 (5.4)	0.63	0.174
	Other		4 (4.3)	0 (0)	0 (0)	0.486	0.486
Respiratory system (ATC R)			2 (2.2)	0 (0)	0 (0)	0.93	0.93
	Drugs for obstructive airway diseases		2 (2.2)	0 (0)	0 (0)	0.93	0.93
Other			1 (1.1)	1 (1.1)	2 (2.2)	1.00	1.00

[a]Chi-square tests were used to test hypotheses, followed by Bonferroni correction, regarding differences in the proportions of patients with TSC and the proportions of patients with IGE and FE.

*P<0.05, **P<0.01, and ***P<0.001 between patients with TSC and IGE; †P<0.05,††P<0.01, and †††P<0.001 between patients with TSC and FE.

Due to the possibility of multiple drug intake per patient in the categories, the number of patients in the subordinated categories may exceed the number of patients in the superordinated categories.

TSC, tuberous sclerosis complex; IGE, idiopathic generalized epilepsy; FE, focal epilepsy; ATC, anatomical therapeutic chemical classification; ASM, anti-seizure medication; mTOR, mechanistic target of rapamycin.
